# Adventitious Agents and Smallpox Vaccine in Strategic National Stockpile

**DOI:** 10.3201/eid1107.050277

**Published:** 2005-07

**Authors:** Frederick A. Murphy, Bennie I. Osburn

**Affiliations:** * University of California, Davis, California, USA

**Keywords:** Keywords: Smallpox, Variola, Vaccine, Adventitious Agents, Strategic National Vaccine Stockpile, Zoonotic transmission, Biodefense

## Abstract

In keeping with current standards, we urge that old smallpox vaccines that were made in animal skin and are still a key part of our strategic national stockpile be tested for adventitious infectious agents. The advisory especially applies to viruses that have the potential for zoonotic transmission to human vaccine recipients.

As we studied recent papers on the manufacture and testing of new smallpox vaccine stocks produced for biodefense purposes, we were surprised that the largest part of our national vaccine stockpile, the Wyeth vaccine Dryvax produced in 1980–1982 and the Connaught (now Aventis-Pasteur) vaccine Wetvax produced in the 1950s, has never been scrutinized by modern methods. Of particular concern is the fact that these stocks have never been subjected to testing for adventitious agents, whereas a new vaccine intended to supplement the existing stockpile has been thoroughly tested (1). Testing of these old stocks met the standards of the day. However, if these old vaccines are to be considered valid parts of our national stockpile we should expect not only continuing testing of potency and sterility but also testing for adventitious agents with methods that reflect the standards of today.

This is not to say that the finding of adventitious agents must result in removal of these vaccine stocks. That issue must be a matter for formal risk analysis and consideration by the same experts who review data on new vaccines (e.g., the Advisory Committee on Immunization Practices of the Centers for Disease Control and Prevention) and by officials of the Food and Drug Administration (FDA). However, as greater concern emerges about the potential pathogenicity of infectious agents that might be present in old vaccine stocks, prudence dictates caution and testing.

Such concerns are amplified by the memory of how these old smallpox vaccines were made. Such vaccines were made in the skin of calves and sheep, and seeds and stocks were passaged in tissues of calves, sheep, and rabbits (especially used for seed lot production). Equally important is the fact that for many decades preceding the development of standardized manufacturing methods in the 20th century, the vaccine virus (vaccinia virus) was propagated by serial passages in animals without precise knowledge of the passage history and without use of a seed lot system that stabilized passage level. This uncontrolled system could have allowed amplification of any passenger viruses and could have increased the possibility of untoward changes in their genetic makeup. In addition, since the crude manufacturing methods (Figure) allowed direct contact of the materials harvested from animals with human operators, possibilities existed for contamination of the resulting product with pathogenic human viruses. It was not uncommon practice before the widespread acceptance of vaccine manufacture in animals (at the end of the 19th century) to passage vaccinia virus arm-to-arm between humans (2). Standardized methods for manufacture in animal skin were not initiated until 1925.

**Figure:**
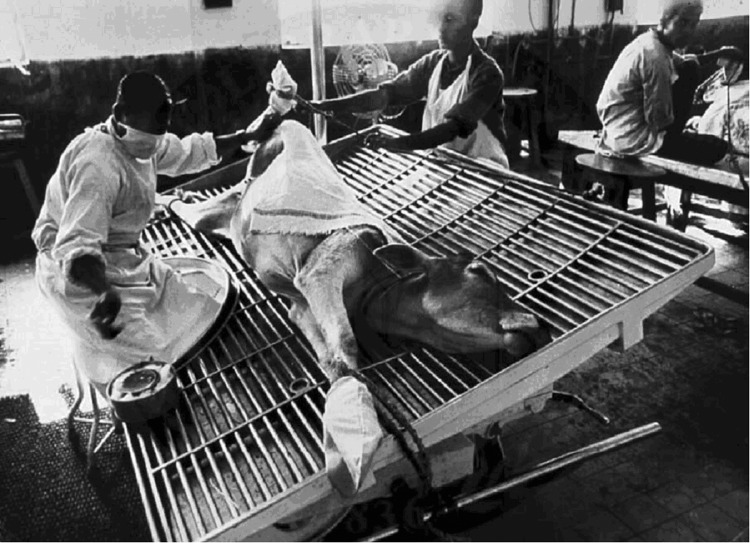
Freeze-dried (smallpox) vaccine being prepared from virus grown on the skin of a calf. The calf is lying on a grate on a table and is bound to the table. Two men in white coverups, 1 of whom has a surgical mask on his face, are performing a procedure on the calf (scarification and introduction of vaccinia virus into the scarified areas). From the record of the US National Library of Medicine; old negative no. 83-168. WHO/11683 SEARO, Smallpox, Bangladesh, SM 5-1 980. Photograph attributed to J. Mohr, 1980?

Such concerns are further amplified by infectious agents that are targeted by modern vaccine testing protocols. When materials of animal skin origin are used, such agents are *Brucella* spp., *Mycobacterium* spp., *Bacillus anthracis*, *Clostridium* spp., and other bacteria and fungi. Old stocks of vaccine were tested for microbes, and the presence of specific pathogenic species was the basis for rejection of vaccine lots. The specifications for approval of dermal vaccines produced in calves and sheep allowed for the presence of a low concentration of nonpathogenic bacteria and fungi.

However, the dermal vaccines produced from the 1950s to the 1980s and currently in the national stockpile were not tested for mycoplasma or viruses. In the case of vaccines manufactured in calves, the agents of concern include several bovine viruses (bovine viral diarrhea virus, bovine parainfluenza virus type 3, bovine respiratory syncytial virus, bovine adenoviruses, bovine parvovirus[es], bovine herpesvirus 1 [infectious bovine rhinotracheitis virus], other bovine herpesviruses, bovine reovirus[es], rabies virus, bluetongue viruses, bovine polyoma virus, bovine circovirus, Cache Valley virus, and orthopoxviruses other than vaccinia [such as cowpox]). We were unable to find a comprehensive list of possible adventitious agents when ovine materials are used, as is the case for the Lister strain smallpox vaccine produced in Europe and old vaccine stocks held by some European countries for biologic defense. Sheep harbor several members of the same virus groups found in cattle, but they also carry other viruses. Rabbits were sometimes used for intermediate passaging of vaccinia virus stocks and for seed virus production, particularly in Europe. Possible rabbit viruses that could contaminate vaccinia stocks include endogenous retroviruses, papillomavirus, herpesviruses, and leporipoxviruses. Because of research and development of specific pathogen-free swine as special organ and tissue donors for human xenotransplantation over the past decade, the list of possible adventitious agents in materials derived from swine is quite comprehensive (3).

When materials of human origin are used, adventitious agents include HIV-1 and HIV-2; human T cell lymphotropic virus type I (HTLV-I) and HTLV-II; hepatitis A, B, and C viruses; human cytomegalovirus; Epstein Barr virus; human herpesviruses 6, 7, and 8; human parvovirus B19; reoviruses; polyoma (JC/BK) viruses; 5V40 virus; human coronaviruses; human papillomaviruses; influenza A, B, and C viruses; various human enteroviruses; human parainfluenza viruses; and human respiratory syncytial virus.

As mentioned, there is a risk that a human virus could have been introduced into smallpox vaccine seed or vaccine stocks during manufacturing, since barrier methods such as those currently used in all phases of vaccine production were not in place. Although the ability of such human viruses to be propagated in subsequent vaccine lots is uncertain, many human viruses are capable of replicating in animal cells.

When materials from any animal source are used, special consideration is given to exogenous and endogenous retroviruses (e.g., bovine immunodeficiency virus), lymphocytic choriomeningitis virus, adenoassociated viruses, minute virus of mice, and other viruses that are notorious contaminants. However, these special considerations fail to include many infectious agents that should raise concern. However, for old smallpox vaccine stocks, it is enough to question whether any of the infectious agents specifically cited in FDA and European Commission regulations, recommendations, and guidelines are present.

Current regulations, recommendations, and guidelines on testing for adventitious microbial and viral agents from various national and international agencies require nonspecific screening and relevant specific tests. Regulations requiring tests for mycoplasma and viruses came into effect long after old stocks of smallpox vaccine were manufactured. Nonspecific screening tests include classic culture tests for bacteria and fungi (sterility tests), special culture and animal tests for *Mycobacterium* spp., cell culture tests for the presence of certain cytocidal viruses (by inoculation of and blind passage in Vero, MRC-5, HeLa, RK, and A9 cells with observations for cytopathology and tests for hemadsorption and hemagglutination at the end of the culture period), and animal inoculation tests for certain viruses (suckling and adult mice, guinea pigs, and embryonated hen eggs). Electron microscopy is often used to find adventitious agents in cell culture banks.

In the United States, federal regulations specify that products of bovine origin (such as virus preparations, cell lysates, cultured cells, or other reagents) intended for use in the production of human biologics be tested for the presence of specific bovine viruses in accordance with 9 C.F.R. 113.53. Specific tests for adventitious infectious agents are conducted using various polymerase chain reaction (PCR)– and immunochemical–based assays. The extraordinary sensitivity of these assays has served to raise the bar of expectation of test veracity, while improving practicality and containing costs. In many cases, these assays have been validated, that is, proof-tested using salted vaccine and vaccine substrate materials. Since companies exist that conduct these specific tests for vaccine developers and manufacturers, such testing on old smallpox vaccine stocks is eminently feasible. The new cell culture-based smallpox vaccine has been tested by using these methods (1). However, considering more advanced PCR-based tests for unknown or unrecognized adventitious agents (e.g., representational difference analysis, use of various degenerate primers) would require extensive research and add substantially to overall costs. For the purpose at hand, we are suggesting only that the battery of specific tests now used on all modern vaccine materials be used.

Concerns about the possible presence of adventitious agents in old smallpox vaccine stocks are amplified further by current concerns about prions and the zoonotic potential of prion diseases. Old smallpox vaccine stocks might not be contaminated by bovine spongiform encephalopathy (BSE) prions, but Lister vaccine stocks that were produced in sheep and vaccine seeds that had been passaged in sheep could be contaminated by scrapie prions. Regulations and guidelines for modern vaccines state that all materials used must come from BSE-free regions but say nothing about scrapie-free regional status. Testing of old vaccines for prions is beyond the sensitivity of any present in vitro prion test, but this issue should be considered (4).

Since no problems related to contamination have been recognized during the long history of smallpox vaccines, or during the intensified program to eradicate smallpox, one might argue that little risk for humans is posed from adventitious agents in old stocks of vaccine. However, it is unlikely that low-incidence untoward events temporally related to adventitious agents have been recognized. It is equally unlikely that diseases that appeared at long intervals after smallpox vaccination would have been associated with the vaccine. Furthermore, since most smallpox vaccine was used in children, we may have less data on its use in adults than we would want. Of note is the recent observation that myopericarditis is a relatively common serious adverse event following smallpox vaccination, but that this complication was not recognized during the era of routine vaccination (5).

Today, the senior guiding document for manufacturers of first-generation smallpox vaccines, i.e., vaccines produced in animal skin, is the World Health Organization (WHO) Recommendations for Production and Control of Smallpox Vaccine, revised 2003 (6). A definitive version of this document will be published in the WHO Technical Report Series (the working version is available from http://www.who.int/biologicals/areas/vaccines/smallpox/en/). FDA documents on this subject are much more general and say little or nothing about adventitious agents (7). The WHO document represents continuation of a series that started in 1956. Several points from the 2003 WHO document (6) are of particular interest here (the chosen points are not meant to be comprehensive or reflect the overall sense of this document).

First, adventitious agent testing for viruses in vaccine virus seeds and product intermediates is complicated and might give ambiguous results. Therefore, newer, more specific tests are planned for the future. Second, testing for viral adventitious agents of animal skin vaccine should take into consideration the source country of the animals. Guidelines for transmissible spongiform encephalopathy testing should be followed. Third, the concentration of nonpathogenic bacteria and fungi in vaccines produced in animal skin may be very difficult to validate, and consistent sterility of the finished product may be difficult to achieve. The use of a nonsterile final product may be justified since smallpox vaccine is administered by scarification rather than by intramuscular or intravenous inoculation, and because its use over many years did not cause problems. Fourth, the general method for testing a live viral vaccine strain for contaminating viruses is to neutralize the vaccine virus and test for adventitious viruses both in vitro and in vivo. However, it is recognized that vaccinia virus is very difficult to neutralize to the extent required for such studies. Additional testing such as nucleic acid amplification techniques for specified viruses and reverse transcriptase assays for retroviruses should complement nonspecific tests. Fifth, in preparing master seed lots, procedures should be used that help remove extraneous agents. Since removal or inactivation of microbial contaminants is unlikely at any downstream level, the presence of extraneous agents in seed lots during the production process must be avoided. Sixth, the absence of specific human pathogens should be confirmed by additional testing, e.g., bacterial and fungal cultures, virus culture, PCR testing for viral agents.

Taken together, these points from the 2003 WHO document make it clear that members of the WHO Expert Committee on Biological Standardization had difficulty dealing with the exceptional problems posed by the first-generation smallpox vaccines, i.e., vaccines produced in animal skin. Their difficulty in producing guidelines also pertained to old lots of such vaccines, such as those that are part of the US and European strategic stockpiles. This seemed most obvious regarding testing for adventitious agents. The limits of such testing seem clear, but so are the practicalities. Standard testing for adventitious agents is practicable and would provide important evidence for risk-benefit considerations if or when old vaccines are used in an emergency situation. Time will eventually obviate such considerations as modern smallpox vaccines replace old vaccines in national stockpiles, but for the present we see the WHO document as another basis for suggesting comprehensive testing of old vaccines.

Since old smallpox vaccine stocks have been in the public domain for many years, we would expect that comprehensive testing would be funded by the same public agency (the US Department of Health and Human Services) that intends to distribute the vaccines should the need arise. We believe that the testing should be fully transparent, that is, fully open to public scrutiny.
